# Does hypertension exacerbate the age‐related exaggerated pressor response to dynamic exercise during post‐exercise muscle ischemia?

**DOI:** 10.14814/phy2.70365

**Published:** 2025-06-06

**Authors:** Daisuke Hasegawa, Amane Hori, Yukiko Okamura, Kenichi Suijo, Masaki Mizuno, Norio Hotta

**Affiliations:** ^1^ College of Life and Health Sciences Chubu University Kasugai Aichi Japan; ^2^ Japan Society for the Promotion of Science Chiyoda‐ku Tokyo Japan; ^3^ Department of Applied Clinical Research UT Southwestern Medical Center Dallas Texas USA

**Keywords:** arterial baroreflex, central command, exercise pressor reflex, muscle mechanoreflex, muscle metaboreflex

## Abstract

Hypertension is known to augment exercise blood pressure (BP). Aging also potentiates BP response to ischemic dynamic exercise. However, whether hypertension further enhances aging‐induced augmented BP response to ischemic dynamic exercise has not yet been investigated. Therefore, we aimed to test the hypothesis that hypertension exacerbates the pressor response to ischemic dynamic exercise in older adults. The participants were classified into the following two groups: nonhypertensive (NHT, *n* = 13, 60–80 years) and hypertensive (HT, *n* = 10, 61–78 years). We compared the BP responses to very light‐intensity rhythmic handgrip exercise during post‐isometric handgrip exercise muscle ischemia (PEMI) simulated as ischemic dynamic exercise between the HT and NHT groups. Both systolic BP (SBP) and diastolic BP (DBP) responses to the rhythmic handgrip exercise during PEMI in the HT group (∆SBP: 48 ± 18 mmHg and ∆DBP: 28 ± 10 mmHg, *p* = 0.007) were significantly higher than those in the NHT group (∆SBP: 34 ± 17 mmHg and ∆DBP: 20 ± 6 mmHg, *p* = 0.003). Importantly, resting SBP was a significant independent determinant of DBP response to the rhythmic handgrip exercise during PEMI (*β* = 0.412, *p* = 0.047). These results suggest that hypertension further elevates the heightened BP response to ischemic dynamic exercise in older adults.

## 1 INTRODUCTION

It is well known that hypertension augments exercise blood pressure (BP) (Leal et al., 2008; Mitchell, 2017; Mizuno et al., 2011a; Seals et al., 1985; Smith, Williams, et al., 2006). As this augmentation potentiates the risk of cardiovascular events (Al Ghorani et al., 2022; Cho et al., 2012), clarifying the regulatory mechanisms underlying hypertension‐induced augmentation of exercise BP is important and clinically relevant. The incidence of hypertension increases with age (Sierra, 2017). We previously reported that aging increases BP response to ischemic rhythmic exercise (Hasegawa et al., 2021). However, it remains unclear whether hypertension further enhances the aging‐induced augmentation of the BP response to ischemic dynamic exercise.

In daily life, various physical activities may induce ischemia‐like conditions, including carrying heavy bags or groceries for an extended period and ascending long flights of stairs without breaks. These activities frequently involve sustained muscle contractions, elevated intramuscular pressure, or external compression, which are believed to transiently restrict perfusion. Recently, physical exercise therapies that actively cause ischemia, such as blood‐flow‐restricted dynamic exercise, have been applied to older adults (Centner et al., 2019). Thus, it is clinically prudent to understand the potential influence of hypertension on BP response to ischemic dynamic exercise in older adults.

The following three autonomic neurocirculatory control mechanisms determine the cardiovascular response to exercise: (1) a feed‐forward mechanism, specifically central command, which occurs when the higher center activates both the locomotor and autonomic regions in the brain during exercise (Williamson et al., 2002, 2006); (2) a neural feedback mechanism, known as the exercise pressor reflex (EPR), generated by the activation of metabolically (muscle metaboreflex) and mechanically sensitive (muscle mechanoreflex) thin‐fiber skeletal muscle afferents (Smith, Mitchell, & Garry, 2006; Teixeira et al., 2020); and (3) a homeostatic mechanism, the baroreflex (Raven et al., 1997). Alterations in the functions of the central command (Dombrowski et al., 2018; Liang et al., 2016), EPR (Leal et al., 2008; Mitchell, 2017; Mizuno et al., 2011a; Sausen et al., 2009; Sidhu et al., 2019; Smith, Williams, et al., 2006), and baroreflex (Goldstein, 1983; Laterza et al., 2007) have been suggested to contribute to the hypertension‐induced augmentation of exercise BP. For example, a previous study suggests that the hypertension‐induced higher BP response to exercise is attributed to enhanced metaboreflex sensitivity (Sausen et al., 2009). Hence, we hypothesized that hypertension enhances aging‐induced augmentation in pressor response to ischemic dynamic exercise in older adults. Therefore, this study aimed to compare the pressor response to ischemic rhythmic handgrip exercise in older adults aged ≥60 years with and without hypertension.

## 2 MATERIALS AND METHODS

### 2.1 Participants and sample size calculation

Some participants in the present study overlapped with those in our previous study (Hasegawa et al., 2021). A priori sample size calculation was performed using G* Power 3.1.9.7 (RRID: SCR_013726, Heinrich‐Heine‐Universität, Düsseldorf, Germany). In this study design (number of groups = 2 and number of measurements = 5), a minimum sample size of 22 was required to achieve a power (1 − *β*) of more than 0.80 with an error probability (*α*) of 0.05 and a medium effect size (Cohen, 1992), using a repeated measure two‐way analysis of variance (ANOVA).

Based on a previous study (Zhang et al., 2020), we defined older adults as those aged ≥60 years. Twenty‐four older adults participated in the study. One participant was excluded from the analysis due to smoking history, resulting in a final sample size of 23. The participants were classified into two groups according to the diagnostic criteria for hypertension (systolic BP (SBP) >140 mmHg or diastolic BP (DBP) >90 mmHg) (Umemura et al., 2019), using resting BP measurements obtained before the exercise test. The groups were as follows: nonhypertensive (NHT, *n* = 13; four males, nine females; aged 60–80 years) and hypertensive (HT, *n* = 10; seven males, three females; aged 61–78 years). Data from older adult participants in our previous study (Hasegawa et al., 2021), excluding the smokers mentioned above, were used for the NHT group in the present study. None of the participants had a history of antihypertensive medication use, kidney disease, peripheral arterial disease, heart failure, peripheral neuropathy, or diabetes, as determined through medical history and medication review during the screening process. These conditions were considered due to their potential influence on exercise BP responses (Grotle et al., 2017; Kim et al., 2019; Park & Middlekauff, 2015; Smith, Mitchell, & Garry, 2006; Stone & Kaufman, 2015).

### 2.2 Ethical approval

This study was approved by the Ethics Committee of Chubu University (No. 290077‐3) and conformed to the principles of the Declaration of Helsinki. The study adhered to the institutional guidelines and regulations for all procedures performed. All participants provided written consent after they were informed about the experimental protocol, including the potential benefits and risks.

### 2.3 Stimuli for evoking pressor response

The exercise stimuli and rationale used in the present study were identical to those used in our previous study (Hasegawa et al., 2021). Briefly, the intensity of the isometric handgrip exercise was set to 30% of the maximum voluntary contraction (MVC) force (Cui et al., 2008; Hotta et al., 2020; Park et al., 2008; Williamson et al., 2002). The participants maintained an appropriate intensity of isometric handgrip exercise for 1 min with visual feedback (Hasegawa et al., 2021; Hotta et al., 2020). Post‐exercise muscle ischemia (PEMI) was induced to observe the BP responses to isolated muscle metaboreflex activation (Batman et al., 1994) by inflating the Hokanson cuff (SC5, Hokanson, Inc., USA) on the left upper arm at a pressure of 250 mmHg using a rapid cuff inflator (E20, Hokanson, Inc., USA) and an air source pump (AG101, Hokanson, Inc., USA) 5 s before the end of the isometric handgrip exercise. During PEMI, the passive wrist movement was performed by flexing and extending the left wrist at a maximum range of motion at 60 cycles per minute to stimulate the mechanoreceptors (Cui et al., 2008; Hotta et al., 2020; Nakamura et al., 2023; Park et al., 2008) under the influence of PEMI‐induced metabolic stimulus. During PEMI, a very low‐intensity rhythmic handgrip exercise was also performed to minimize the influences of the central command (Park et al., 2008) and additional metabolic stimulus (Batman et al., 1994) by instructing the participants to flex and extend their left fingers to the maximum range of motion at a speed of 60 cycles per minute with minimal force (Hotta et al., 2020).

### 2.4 Study design

This study compared BP and heart rate (HR) responses to dynamic exercise during PEMI following isometric exercise, which simulated ischemic dynamic exercise in a way similar to that in our previous study (Hasegawa et al., 2021), between the HT and NHT groups. Several demographic, clinical, and physiological parameters have been reported to affect the cardiovascular responses to exercise, including body mass index (BMI) (Limberg et al., 2018), arterial stiffness (Thanassoulis et al., 2012), MVC force (Lee et al., 2021; Notay et al., 2018; Stavres et al., 2024), and sex (D'Souza et al., 2023; Smith et al., 2019; Tharpe et al., 2023; Trinity et al., 2018). Therefore, a multivariate analysis was conducted to investigate whether resting BP, independent of these parameters, could explain the pressor response to ischemic dynamic exercise.

This study was not blinded. However, since BP and HR measurements were automated (see section 2.6), and the experimenter was unaware of the participants' group assignments, the risk of bias due to the lack of blinding was effectively minimized.

Rhythmic handgrip exercise during PEMI (Hasegawa et al., 2021) was selected as a model of ischemic dynamic exercise instead of the traditional rhythmic handgrip exercise with circulatory arrest (Alam & Smirk, 1937) for the following reasons: First, ischemic dynamic exercise could be performed over a shorter duration because metabolites had already accumulated by the onset of rhythmic handgrip exercise. Consequently, the effects of ischemic dynamic exercise could be captured within a shorter exercise duration. Second, the substantial accumulation of metabolites amplified the BP response, thereby facilitating a clearer assessment of ischemia‐induced cardiovascular effects. Third, to better delineate the underlying mechanisms, we aimed to separately assess the contributions of the muscle mechanoreflex, which may be influenced by metabolite accumulation (Adreani & Kaufman, 1998; Cui et al., 2008; Hotta et al., 2019) (evaluated through passive wrist movement during PEMI), and the muscle metaboreflex (evaluated through the rest period during PEMI).

### 2.5 Experimental protocols

**FIGURE 1:**
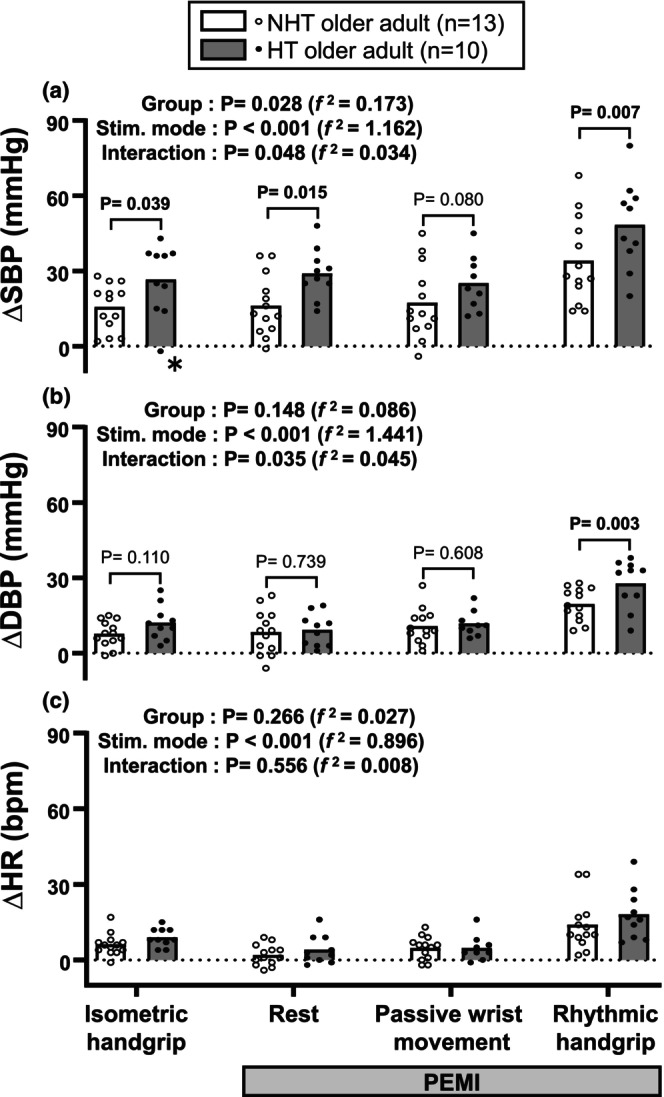
Comparisons of SBP, DBP, and HR responses (Δ) between the hypertensive (HT) and nonhypertensive (NHT) groups from baseline to the isometric handgrip exercise as well as rest, passive wrist movement, and rhythmic handgrip exercise during post‐exercise muscle ischemia (PEMI). DBP, diastolic blood pressure; HR, heart rate; HT, hypertensive; NHT, nonhypertensive; SBP, systolic blood pressure. Data were tested using a linear mixed model. Cohen's *f* ^*2*^ was used as the effect size (0.02, 0.15, and 0.35 for small, medium, and large, respectively). Values are means. The NHT older adults were selected from our previous study (Hasegawa et al., 2021). The baseline values are shown as SBP, DBP, and HR at rest in Table 1. Detailed post hoc comparisons among stimulation modes are provided in Table S1. *The SBP response during isometric handgrip exercise rarely falls below zero (Wakeham et al., 2023), raising the question of whether this occurrence was due to a technical artifact or a genuine physiological response. Although these data exceeded the mean of −1.96 SD, they were not identified as significant outliers according to the Smirnov–Grubbs test (*p* = 0.091). Even if these data were excluded, the statistical results remained unchanged (Group effect: *p* = 0.020 [*f* ^*2*^ = 0.199], Stim mode effect: *p* < 0.001 [*f* ^*2*^ = 1.225], interaction: *p* = 0.036 [*f* ^*2*^ = 0.041], and Post hoc: Isometric handgrip exercise, *p* = 0.015; rest during PEMI, *p* = 0.013; passive movement during PEMI, *p* = 0.075; and rhythmic handgrip exercise during PEMI, *p* = 0.006).

The present study used the same protocol as our previous study (Hasegawa et al., 2021). Before the experiment, the participants rested in a seated position for a sufficient period. After baseline measurements, the participants performed an isometric handgrip exercise on their left arm, followed by a 3‐min protocol for PEMI. During the PEMI, the participants rested for the 1st min, followed by passive wrist movement for the 2nd min, and rhythmic handgrip exercise for the final 3rd min, as indicated on the horizontal axis of Figure 1. All HT participants completed this experiment.

An additional experiment was performed to ensure that the ischemia‐induced elevation in BP remained consistent throughout the 3 min of PEMI, during which the participants rested and did not perform either the passive wrist movement or rhythmic handgrip exercise. The time points were defined as PEMI 1st min, PEMI 2nd min, and PEMI 3rd min. Eight of the 10 participants in the HT group participated (*n* = 8) and completed this experiment.

Laboratory temperatures were maintained at approximately 25°C. We instructed the participants to abstain from alcohol, caffeine, and prolonged and/or high‐intensity exercise the day before the experiment. On the day of the experiment, the participants were prohibited from consuming any food or beverages, except for water, for 2 h before the experiment.

### 2.6 Measurements

The measurements in the present study were similar to those used in our previous study (Hasegawa et al., 2021). Before the exercise test, using a semiautomated vascular screening system (Vasera 1500 N, Fukuda Denshi, Japan), the cardio‐ankle vascular index (CAVI) was measured to evaluate arterial stiffness (Shirai et al., 2011). The average left and right CAVI values were used as representative values. We assessed whole‐body fat and muscle mass using a body composition analyzer based on multifrequency bioelectrical impedance analysis (MC‐ 980, Tanita, Japan) (Heymsfield et al., 2015; Kaminska et al., 2018).

During the exercise test, we determined the SBP and DBP on the participant's right upper arm and HR every minute using an automated sphygmomanometer for exercise testing and a standard three‐lead ECG unit (Tango +, Sun Tech Medical Instruments Inc., USA). We used the Borg scale (Borg, 1982) to obtain a participant's perceived exertion (RPE) rating at the end of isometric handgrip and rhythmic handgrip exercises during PEMI to evaluate the sense of effort.

### 2.7 Statistical analysis

We first performed the Shapiro–Wilk test to assess the normality of the data. Since the data followed a normal distribution, an unpaired *t*‐test was used to compare the two groups (NHT vs. HT). Cohen's d was used to measure the effect size of an unpaired *t*‐test; Cohen proposed this effect size classification as 0.2 (small), 0.5 (medium), and 0.8 (large) (Cohen, 1988). Fisher's exact test was used for rate comparisons. Phi (φ) was used as the effect size and is considered with thresholds of 0.1 (small), 0.3 (medium), and 0.5 (large) (Cohen, 1988).

BP and HR responses during the exercise test were analyzed using a linear mixed model, both with and without adjustments for covariates. Effect sizes were assessed using Cohen's *f* ^*2*^, with thresholds of 0.02 (small), 0.15 (medium), and 0.35 (large), as defined by Cohen (Cohen, 1988). The factors included “group” (NHT and HT), “stimulation mode” (isometric handgrip exercise, as well as rest, passive wrist movement, and rhythmic handgrip exercise during PEMI for the main experiment), and “time point” (isometric handgrip exercise, PEMI 1st min, PEMI 2nd min, and PEMI 3rd min for the additional experiment). If an interaction and/or a main effect was significant, Tukey's post hoc test was employed to compare differences between “group” (NHT vs. HT) and within the repeatedly measured factors, namely “stimulus mode” and “time point.”

A two‐factor ANOVA was performed as a subanalysis to address potential confounding variables identified in this study. Effect sizes were assessed using *η*_*p*_^2^, with thresholds of 0.01 (small), 0.06 (medium), and 0.14 (large) (Cohen, 1988). Factor 1 was “group” (NHT vs. HT), and factor 2 was stratified based on sex (female vs. male) or higher versus lower values of the relevant index. For stratification based on higher versus lower values of the relevant index, participants were categorized according to the median value of the index. When a significant interaction was detected, Tukey's test was employed for post hoc analysis to compare group differences (NHT vs. HT).

Relationships between BP responses to the exercise test and demographic, clinical, and physiological parameters were assessed using Spearman's or Pearson's correlations. Point‐biserial correlation analysis was employed to analyze the relationship with sex, a categorical variable. Pearson's partial correlation coefficients were calculated after adjusting for a set of covariates. To identify significant determinant factors of the BP responses to ischemic dynamic exercise, multivariable‐adjusted linear regression analyses were conducted using the forced entry method. Parameters that significantly correlated with BP response indices were included in the regression model. Age and sex were included a priori as covariates, given the established sex differences in BP regulation during exercise among older adults (D'Souza et al., 2023; Smith et al., 2019; Trinity et al., 2018). BMI was included as an additional covariate because of its known influence on BP responses during exercise (Limberg et al., 2018); the corresponding results are presented in the Results section. To assess potential multicollinearity among parameters, the variance inflation factor was calculated, with values exceeding 5.0 indicating significant collinearity.

Data are expressed as mean ± standard deviation (SD). Statistical analyses were performed using R‐ 4.4.2 (RRID:SCR_001905, free software), GraphPad Prism 8 (RRID SCR_002798, GraphPad Software Inc., San Diego, CA, USA), and IBM SPSS Statistics for Windows, version 28.0 (RRID: SCR_002865, IBM Corp., Armonk, NY, USA). The significance level for all tests was set at *α* < 0.05.

## 3 RESULTS

Participants' characteristics are presented in Table 1. Body mass, BMI, and MVC force in the HT group were significantly higher than those in the NHT group. The effect size for BMI was large and was the greatest among all variables, except for resting BP indices. Although the MVC force in the HT group was also significantly greater than that in the NHT group, the MVC force per body mass was not significantly different between the two groups. As a matter of course, resting SBP and DBP in the HT group were significantly higher than those in the NHT group.

**TABLE 1:** Participant characteristics.

	NHT older adult (*n* = 13)	HT older adult (*n* = 10)	*p* Value	d
	Mean ± SD	Mean ± SD		
Age years	69 ± 7	68 ± 5	0.807	0.104
	(60–80)	(61–78)		
Female/male ratio %	9/4 (69/31 %)	3/7 (30/70 %)	0.100^a^	0.389^b^
Height cm	157.3 ± 7.6	161.9 ± 10.7	0.240	0.508
Body mass kg	52.5 ± 5.7	63.4 ± 9.1	0.002	1.487
BMI kg/m^2^	21.2 ± 2.0	24.1 ± 1.3	0.001	1.660
Body fat percentage %	24.7 ± 6.3	25.8 ± 7.1	0.682	0.175
Muscle rate %	71.2 ± 6.1	70.3 ± 6.8	0.722	0.152
CAVI	8.6 ± 0.8	8.8 ± 0.4	0.509	0.283
MVC *N*	232 ± 66	315 ± 109	0.033	0.957
MVC/body mass *N*/kg	4.4 ± 1.0	4.9 ± 1.2	0.289	0.458
SBP rest mmHg	116 ± 9	147 ± 14	<0.001	2.675
DBP rest mmHg	77 ± 7	89 ± 10	0.006	1.281
HR rest bpm	66 ± 7	63 ± 9	0.464	0.314

*Note*: Data were tested using an unpaired *t*‐test. Cohen's *d* was used as the effect size (0.2, 0.5, and 0.8 represent small, medium, and large, respectively). The NHT older adults were selected from our previous study (Hasegawa et al., 2021).

Abbreviations: BMI, body mass index; CAVI, cardio‐ankle vascular index; DBP, diastolic blood pressure; HR, heart rate; HT, hypertensive; NHT, nonhypertensive; MVC, maximum voluntary contraction; SBP, systolic blood pressure.

^a^ *p* Value was achieved using Fisher's exact test.

^b^ Phi was used as the effect size (0.1, 0.3, and 0.5 for small, medium, and large, respectively).

We first assessed whether the ischemia‐induced elevation in BP remained consistent throughout the 3 min of PEMI in both groups (Table 2). Regardless of the group, PEMI significantly elevated BP, and BP exhibited no significant change during the 3 min of PEMI.

**TABLE 2:** SBP, DBP, and HR responses (Δ) from baseline to isometric handgrip exercise and 3‐min of PEMI.

		*n*	Isometric handgrip		PEMI						*p* Value (*f* ^*2*^)		
					1st min		2nd min		3rd min		Group	Time point	Interaction
ΔSBP (mmHg)	NHT	13	15 ± 10	*	15 ± 11	*	13 ± 10	*	12 ± 9	*	0.088	<0.001	0.088
	HT	8	29 ± 18		23 ± 17		20 ± 17		23 ± 18		(0.126)	(0.400)	(0.030)
ΔDBP (mmHg)	NHT	13	8 ± 4	*	6 ± 5	*	8 ± 3	*	8 ± 5	*	0.747	<0.001	0.754
	HT	8	10 ± 5		7 ± 3		7 ± 3		8 ± 3		(−0.005)	(0.672)	(0.002)
ΔHR (bpm)	NHT	13	7 ± 4	*	0 ± 2		2 ± 2	*	3 ± 3	*	0.081	<0.001	0.115
	HT	8	7 ± 3		4 ± 4		6 ± 6		5 ± 2		(0.109)	(0.554)	(0.043)

*Note*: Data were tested using a linear mixed model. Cohen's *f* ^*2*^ was used as the effect size (0.02, 0.15, and 0.35 represent small, medium, and large, respectively). Tukey's test was employed for post hoc analysis. The NHT older adults were selected from our previous study (Hasegawa et al., 2021). Asterisks indicate significant differences from baseline, regardless of the group (*p* < 0.001). The baseline values in the NHT groups are presented in Table 1 as SBP, DBP, and HR at rest. Those in the HT group were 144 ± 12 mmHg, 90 ± 9 mmHg, and 64 ± 9 bpm, respectively (eight of the 10 individuals participated in this additional experiment). Data are expressed as mean ± standard deviation.

Abbreviations: DBP, diastolic blood pressure; HR, heart rate; HT, hypertensive; NHT, nonhypertensive; PEMI, post‐exercise muscle ischemia; SBP, systolic blood pressure.

Figure 1 shows the main outcomes of this study, specifically BP and HR responses from baseline to the isometric handgrip exercise, as well as rest, passive wrist movement, and rhythmic handgrip exercise during PEMI in the NHT and HT groups. One participant in the HT group had data not available for passive wrist movement. The main effects of the stimulation mode were significant in the BP and HR responses. Additionally, all responses except for the HR response to rest and passive wrist movement during PEMI were significantly increased compared with the baseline, regardless of the group (Table S1). During PEMI, regardless of the group, BP and HR responses to the rhythmic handgrip exercise were significantly higher than those at rest (*p* < 0.0001); however, no significant differences were detected between rest and passive wrist movement (*p* > 0.528). Importantly, significant group‐by‐stimulation mode interactions were found in SBP and DBP responses. Post hoc analysis revealed that SBP responses to the isometric handgrip exercise, as well as rest and rhythmic handgrip exercise during PEMI, and DBP responses to rhythmic handgrip exercise during PEMI in the HT group were significantly higher than those in the NHT group.

The results presented in Table 1 suggest that BMI and MVC force could act as confounding factors in the analysis of BP parameters shown in Figure 1. Multiple statistical models were employed to address these and other potential confounders. Age and sex were also incorporated as potential covariates in the analysis. Table 3 summarizes the *p* values and effect sizes for the main effects of group and group‐by‐stimulation mode interactions, both before and after adjusting for covariates. The unadjusted (crude) model served as a baseline reference, as illustrated in Figure 1. In the first adjusted model (Model 1), which included resting BP as a covariate, the observed significant group effects and interactions in SBP and DBP responses were eliminated. This finding confirmed that resting BP was a key confounder, consistent with the study's foundational premise. Model 2 included MVC force as a covariate. While the SBP response remained significant compared to the crude model, the DBP response lost significance. From Models 3 to 5, additional adjustments were sequentially applied for demographic (age and sex) and clinical (BMI) characteristics. For the SBP response, statistical significance was lost upon adding BMI as a covariate. Regarding the DBP response, statistical outcomes were influenced by adjustments for either sex or BMI. These results highlighted the sensitivity of the BP parameters to confounding factors, including not only resting BP but also MVC force, sex, and BMI.

**TABLE 3:** *p* Values and effect sizes of unadjusted and adjusted models for the main effect of group (HT and NHT), the interaction, and post hoc analyses.

	Adjuster		Group effect	Interaction	Post hoc test (HT vs. NHT)			
					Isometric handgrip	Rest	Passive wrist movement	Rhythmic handgrip
						PEMI		
SBP response								
Crude model	Unadjusted	*P*	**0.028**	**0.048**	**0.039**	**0.015**	0.080	**0.007**
		*f* ^*2*^	0.173	0.034				
Model 1	SBP rest and DBP rest	*P*	0.452	0.792	–	–	–	–
		*f* ^*2*^	−0.002	−0.001				
Model 2	MVC	*P*	**0.039**	**0.038**	0.115	**0.011**	0.111	**0.007**
		*f* ^*2*^	0.161	0.037				
Model 3	Age	*P*	**0.030**	**0.049**	**0.041**	**0.018**	0.082	**0.007**
		*f* ^*2*^	0.174	0.034				
Model 4	Age and sex	*P*	**0.012**	**0.003**	0.055	**0.004**	**0.031**	**0.001**
		*f* ^*2*^	0.275	0.065				
Model 5	Age, sex, and BMI	*P*	0.125	0.251	–	–	–	–
		*f* ^*2*^	0.074	0.013				
DBP response								
Crude model	Unadjusted	*P*	0.148	**0.035**	0.110	0.739	0.608	**0.003**
		*f* ^*2*^	0.086	0.045				
Model 1	SBP rest and DBP rest	*P*	0.601	0.326	–	–	–	–
		*f* ^*2*^	0.006	0.014				
Model 2	MVC	*P*	0.365	0.499	–	–	–	–
		*f* ^*2*^	0.011	0.005				
Model 3	Age	*P*	0.165	**0.035**	0.122	0.811	0.620	**0.004**
		*f* ^*2*^	0.083	0.046				
Model 4	Age and sex	*P*	0.252	0.407	–	–	–	–
		*f* ^*2*^	0.028	0.008				
Model 5	Age, sex, and BMI	*P*	0.841	0.844	–	–	–	–
		*f* ^*2*^	−0.024	−0.006				

*Note*: The unadjusted (crude) model served as a baseline reference, as illustrated in Figure 1. Data were tested using a linear mixed model with covariate adjustment. Cohen's *f* ^*2*^ was used as the effect size (0.02, 0.15, and 0.35 represent small, medium, and large, respectively). Tukey's test was employed for post hoc analysis. Significant values are indicated in bold font. The NHT older adults were selected from our previous study (Hasegawa et al., 2021).

Abbreviations: BMI, body mass index; DBP, diastolic blood pressure; HT, hypertensive; MVC, maximal handgrip strength; NHT, nonhypertensive; SBP, systolic blood pressure.

We conducted a stratified subanalysis to evaluate the potential moderating effects of MVC force, sex, and BMI on the group differences in BP response parameters observed in Figure 1. Participants were categorized based on sex, BMI (higher vs. lower groups), and MVC force (higher vs. lower groups) (Figure S1). The stratified analyses based on sex and BMI revealed no group‐by‐subgroup interactions in any BP response indices (Figure S1a–h). However, a significant group (HT vs. NHT)‐by‐MVC force (lower vs. higher) interaction was observed in the SBP response to rest during PEMI (Figure S1j). Post hoc analysis showed that the SBP response was significantly higher in the HT group than in the NHT group, but only within the higher MVC force subgroup. Similarly, while the interaction was not statistically significant, a trend was noted in DBP response to rhythmic handgrip during PEMI (Figure S1l), with visually greater BP response in the HT group than in the NHT group, which again was confined to the higher MVC force subgroup.

The evaluation of volitional effort sense during exercise revealed that neither RPE for the isometric handgrip exercise nor rhythmic handgrip exercise during PEMI was significantly different between the NHT (12 ± 2 and 14 ± 2, respectively, *n* = 13) and HT (12 ± 2 and 16 ± 2, respectively, *n* = 10) groups (*p* = 0.708 and 0.125, d = 0.160 and 0.672, respectively).

Table 4 presents the results of the single correlation analyses between demographic, clinical, and physiological parameters and cardiovascular values that were significantly different between the NHT and HT groups in Figure 1, such as SBP response to the isometric handgrip exercise, as well as rest during PEMI, and SBP and DBP responses to the rhythmic handgrip exercise during PEMI. No indices demonstrating a significant correlation with the SBP response to the isometric handgrip exercise were detected. SBP responses to rest and rhythmic handgrip exercise during PEMI and DBP responses to rhythmic handgrip exercise during PEMI were significantly positively correlated with resting SBP. Significant positive correlations were also detected between SBP response to rhythmic handgrip exercise during PEMI and BMI and between DBP response to rhythmic handgrip exercise during PEMI and MVC force.

**TABLE 4:** Results of single correlation analysis between blood pressure responses to the isometric handgrip exercise, and rest and rhythmic handgrip exercise during post‐exercise muscle ischemia (PEMI) and demographic, clinical, and physiological variables.

Variables	Mean	SD	SBP response						DBP response	
			Isometric handgrip		Rest during PEMI		Rhythmic handgrip during PEMI		Rhythmic handgrip during PEMI	
			*r*	*p*	*r*	*p*	*r*	*p*	*r*	*p*
SBP rest mmHg	129	19	0.354	0.098	**0.474**	**0.022**	**0.416**	**0.048**	**0.472**	**0.023**
DBP rest mmHg	82	10	0.305	0.157	0.302	0.161	0.263	0.226	0.152	0.489
Age years	69	6	0.050	0.821	−0.034	0.878	0.143	0.515	−0.077	0.727
Sex	Female = 0 (52.2%), male = 1 (47.8%)		0.192^rpb^	0.380	−0.099^rpb^	0.654	−0.122^rpb^	0.578	0.411^rpb^	0.052
BMI kg/m^2^	22.5	2.2	0.306	0.156	0.384	0.071	**0.524**	**0.010**	0.400	0.058
CAVI	8.7	0.6	0.395	0.062	0.189	0.388	0.007	0.976	0.222	0.309
MVC *N*	268	95	0.308	0.153	0.048	0.830	0.075	0.735	**0.532**	**0.009**
RPE isometric handgrip	12.1	1.8	−0.067	0.762	−0.006	0.977	−0.229	0.293	−0.045	0.838
RPE rhythmic handgrip during PEMI	14.9	2.3	0.279	0.197	0.324	0.131	0.254	0.242	0.114	0.606

*Note*: Pearson's (*r*) correlation coefficients were used depending on the data distribution. The relationships with sex (a categorical variable) were tested using point‐biserial correlation (*r*_pb_) analysis. Significant *p* values are indicated in bold font. Blood pressure response indices that showed significant group differences in Figure 1 were chosen. *n* = 23.

Abbreviations: BMI, body mass index; CAVI, cardio‐ankle vascular index; DBP, diastolic blood pressure; MVC, maximal handgrip strength; RPE, rating of perceived exertion; SBP, systolic blood pressure.

A key finding in Table 4 highlights the significant positive correlation between SBP at rest and three indices of BP response: the SBP response to rest during PEMI, the SBP response to rhythmic handgrip during PEMI, and the DBP response to rhythmic handgrip during PEMI. To further examine these associations while accounting for potential confounding factors, partial correlation analyses were conducted (Table 5) using the same covariate adjustment models described in Table 3. The unadjusted (crude) model served as a baseline reference, corresponding to the SBP rest row in Table 4. The correlation between SBP at rest and the SBP response to rest during PEMI remained largely unchanged after adjusting for covariates, suggesting that this relationship was not substantially confounded. In contrast, associations between SBP at rest and both SBP and DBP responses to rhythmic handgrip during PEMI were attenuated, particularly when BMI was included as a covariate. These findings suggest that BMI may have served as a significant confounding factor in relationships between SBP at rest and BP response to rhythmic handgrip exercise during PEMI.

**TABLE 5:** Partial correlation analysis between resting systolic blood pressure (SBP) and blood pressure response indices.

	Adjuster	SBP response						DBP response	
		Isometric handgrip		Rest during PEMI		Rhythmic handgrip during PEMI		Rhythmic handgrip during PEMI	
		*r*	*p*	*r*	*p*	*r*	*p*	*r*	*p*
Crude model	Unadjusted	0.354	0.098	**0.474**	**0.022**	**0.416**	**0.048**	**0.472**	**0.023**
Model 1	MVC	0.302	0.173	**0.478**	**0.025**	0.412	0.057	0.415	0.055
Model 2	Age	0.351	0.109	**0.491**	**0.020**	0.400	0.065	**0.498**	**0.018**
Model 3	Age and sex	0.312	0.168	**0.557**	**0.009**	**0.493**	**0.023**	0.412	0.064
Model 4	Age, sex, and BMI	0.222	0.346	**0.454**	**0.044**	0.328	0.158	0.297	0.203

*Note*: Partial correlation coefficients (Pearson's *r*) were calculated after adjusting for a set of covariates, including demographic, clinical, and physiological variables, using the same covariate models as in Table 3. The unadjusted (crude) model served as a baseline reference and corresponds to the SBP rest row in Table 4. Statistically significant *p* values are indicated in bold.

Abbreviations: BMI, body mass index; DBP, diastolic blood pressure; MVC, maximal handgrip strength.

Finally, we performed multivariable‐adjusted linear regression analyses using the parameters significantly correlated with BP response indices, specifically SBP at rest, BMI, and MVC force in Table 4. Although age and sex were not significantly correlated with any BP response indices, they were included a priori as covariates because, as indicated in Table 3, sex had been suggested to have acted as a confounding factor. Importantly, as presented in Table 6, resting SBP was a significant independent determinant of the DBP response to the rhythmic handgrip exercise during PEMI. Resting SBP was also a significant independent determinant of SBP response to rest during PEMI. However, resting SBP was not a significant independent determinant of the SBP response to rhythmic handgrip exercise during PEMI.

**TABLE 6:** Multivariable‐adjusted linear regression models.

Variables included in the model		*β*	*R* ^2^	*p* Value
SBP response				
To rest during PEMI	SBP rest	**0.601**	**0.210**	**0.009**
	Sex	−0.303		0.164
	Age	−0.069		0.730
To rhythmic handgrip during PEMI	BMI	**0.446**	**0.299**	**0.045**
	SBP rest	0.317		0.158
	Sex	−0.382		0.069
	Age	0.101		0.596
DBP response				
To rhythmic handgrip during PEMI	SBP rest	**0.412**	**0.301**	**0.047**
	MVC	0.592		0.117
	Sex	−0.207		0.594
	Age	−0.046		0.840

*Note*: The independent variables that were significantly correlated with the systolic blood pressure (SBP) response to post‐exercise muscle ischemia (PEMI) and the SBP and diastolic blood pressure (DBP) responses to the rhythmic handgrip exercise during PEMI in Table 4 were selected for these analyses. Age and sex were incorporated as adjustment factors to account for their potential confounding effects, despite the lack of significant correlations with the BP indices in Table 4. *R* ^2^ and *β* represent the adjusted coefficient of determination and the standardized regression coefficient, respectively. The variance inflation factors of all independent factors were <4.55. Significant values are indicated in bold font. *n* = 23.

Abbreviations: BMI, body mass index; MVC, maximal handgrip strength.

Based on the findings presented in Tables 1 and 5, which indicated the relevance of BMI, we conducted an additional analysis by including BMI as a covariate in models for the SBP response to rest during PEMI and the DBP response to rhythmic handgrip during PEMI, wherein BMI had not initially been considered as an independent variable. The results (Table S2) showed that resting SBP remained a significant independent determinant of the SBP response to rest during PEMI. However, its association with the DBP response to rhythmic handgrip during PEMI was no longer statistically significant, suggesting that BMI may have acted as a confounding factor in this relationship.

## 4 DISCUSSION

Our previous study demonstrated that aging enhances the pressor response to ischemic dynamic exercise (Hasegawa et al., 2021). As a follow‐up to our previous research, this study confirmed that hypertension intensified the SBP response to both isometric handgrip exercise and rest during PEMI, consistent with previous findings (Delaney et al., 2010; Sausen et al., 2009; Seals et al., 1985). In contrast, a novel aspect of this study is the direct comparison of pressor responses to ischemic dynamic exercise between hypertensive and normotensive older adults. Specifically, we found that hypertension significantly augmented both SBP and DBP responses to rhythmic handgrip exercise during PEMI. Moreover, this study identified MVC force, BMI, and sex as potential confounding factors influencing the effects of hypertension on the BP response to rhythmic handgrip exercise. These findings are consistent with previous studies showing that these variables modulate cardiovascular regulation during exercise (D'Souza et al., 2023; Lee et al., 2021; Limberg et al., 2018; Notay et al., 2018; Smith et al., 2019; Stavres et al., 2024; Tharpe et al., 2023; Trinity et al., 2018) and are therefore largely confirmatory. Importantly, however, a key novel finding emerged from the multiple regression analysis: resting SBP was identified as a significant independent determinant of the DBP response to rhythmic handgrip exercise during PEMI. This result highlights the critical role of baseline BP in modulating the pressor response to ischemic dynamic exercise and provides new mechanistic insight. Notably, this association was attenuated when BMI was included as a covariate, suggesting that BMI may also have acted as a meaningful confounding variable and warrants further investigation.

### 4.1 Potential mechanisms that may contribute to the augmented BP response to ischemic exercise in older adults

Hypertension is known to exaggerate the EPR (Leal et al., 2008; Mitchell, 2017; Mizuno et al., 2011a; Smith, Williams, et al., 2006). Studies in humans and animals have reported that hypertension enhances (Delaney et al., 2010; Dombrowski et al., 2018; Sausen et al., 2009), attenuates (Dombrowski et al., 2018), and does not affect (Dombrowski et al., 2018; Rondon et al., 2006; Spranger et al., 2015) the muscle metaboreflex. In the present study, the SBP response to rest during PEMI was significantly higher in the HT group than in the NHT group. This study, along with previous research demonstrating that the PEMI‐induced pressor reflex was higher in older adults than in young adults (Milia et al., 2015), suggests that hypertension further enhances the aging‐induced augmentation of the muscle metaboreflex.

Hypertension has been suggested to enhance the muscle mechanoreflex (Choi et al., 2013; Mizuno et al., 2011b). Additionally, evidence indicates that metabolic by‐products contribute to the augmentation of the muscle mechanoreflex (Adreani & Kaufman, 1998; Cui et al., 2008). However, the combined effects of hypertension and metabolic by‐products on mechanoreflex sensitivity remain unknown. In this study, passive wrist movement was assumed to be performed to minimize the effects of central command and additional muscle metaboreflex activation, although the absence of electromyographic data precludes the direct exclusion of potential muscle contractions. In the present study, no significant group differences were found in the pressor responses to the passive wrist movement during PEMI. Therefore, in this investigation, hypertension may not be involved in sensitizing muscle mechanoreceptors via metabolites associated with PEMI in older adults. Alternatively, the mechanical stimuli generated by the passive wrist movement employed in this study might have been insufficient to detect the effects of mechanoreceptor sensitization. In fact, no significant differences were detected in BP and HR response parameters between rest and passive wrist movement during PEMI in this study. Further study is needed to explore these effects.

The effect of the central command on the circulatory response to exercise is related to the sensation of effort during exercise (Williamson et al., 2006). The RPE did not differ between the HT and NHT groups in this study. Therefore, the effect of the difference in the sense of effort may be small. In this study, the SBP response in the HT group was significantly higher at rest during PEMI, suggesting that hypertension increases the BP response to ischemic dynamic exercise in older adults, even in the absence of augmentation in the central command. However, this implication does not imply that hypertension does not affect central command during ischemic dynamic exercise in older adults, as we did not directly evaluate central command. Actually, the HT group exhibited a greater SBP response to the isometric handgrip exercise, which combined central and peripheral stimuli.

It is well established that experimental concurrent stimulation of the EPR and central command pathways attenuates the pressor response in normotensive rats (Degtyarenko & Kaufman, 2000, 2002; Laurin et al., 2015). In contrast, concurrent stimulation does not significantly affect spontaneously hypertensive rats (Liang et al., 2019). These findings suggest that neural occlusion between peripheral and central mechanisms contributing to the pressor response is diminished in hypertension. Their evidence (Liang et al., 2019) led us to speculate that hypertension may have deteriorated the interactive neural relationship between the EPR and central command, resulting in an enhanced pressor response to the rhythmic handgrip exercise during PEMI in the HT group in this study.

Aging (Carrington & White, 2002) and hypertension (Goldstein, 1983; Laterza et al., 2007) are known to impair baroreflex function. Consequently, even though BP increases during exercise, the effectiveness of negative feedback control is reduced, leading to an exaggerated pressor response to rhythmic handgrip exercise during PEMI in the HT group. Additionally, increased arterial stiffness, commonly observed in older adults (Hasegawa et al., 2021), is a well‐established factor contributing to decreased baroreflex function (Okada et al., 2012). Hypertension, in turn, is an independent determinant of increased arterial stiffness (Zheng et al., 2015). However, in this study, no significant differences were observed in CAVI, an index of arterial stiffness, between the two groups. Therefore, the absence of group differences in CAVI suggests that arterial stiffness, as assessed using this index, does not account for the effect of hypertension on baroreflex function in this study. Nevertheless, since baroreflex function was not directly assessed in this study, this remains speculative.

It is well established that the DBP response is closely associated with muscle sympathetic nerve activity (MSNA) (Ichinose et al., 2014). Previous studies have also demonstrated that the MSNA responses during PEMI are higher in participants with hypertension (Delaney et al., 2010). In this study, the DBP response to ischemic dynamic exercise was significantly greater in the hypertensive group, suggesting that the MSNA response during the ischemic exercise was also heightened in this population. However, as MSNA was not measured in this study, further speculation on this matter is unwarranted.

This study identified not only resting BP but also sex, handgrip strength, and BMI as confounders influencing the effects of hypertension on BP response to exercise. Previous studies have highlighted sex differences among older adults in BP regulation at rest, during exercise, and during ischemia (D'Souza et al., 2023; Smith et al., 2019; Trinity et al., 2018). Additionally, evidence suggests that absolute handgrip strength and BMI affect BP responses to isometric handgrip exercise (Lee et al., 2021; Limberg et al., 2018; Notay et al., 2018; Stavres et al., 2024). These findings underscore the importance of considering such confounding factors when evaluating the primary outcomes of this study. Notably, the subanalysis suggests that hypertension‐induced exaggeration of the DBP response to ischemic dynamic exercise is more pronounced in individuals with relatively higher muscle strength. However, acknowledging that stratified analyses based on sex, handgrip strength, and BMI are limited by the small sample size and should be interpreted as exploratory is important. Further studies with larger cohorts are warranted to confirm these findings. Most importantly, our multiple regression analysis demonstrated that resting SBP was an independent determinant of the DBP response to ischemic rhythmic handgrip exercise. This key finding strongly supports our hypothesis that hypertension further amplifies the aging‐induced heightened BP response to ischemic dynamic exercise. Nevertheless, the potential confounding influence of BMI should not be overlooked and warrants consideration in future investigations.

Exercise DBP depends, at least partially, on the resting DBP (Tuka et al., 2015). Surprisingly, the resting SBP was a significant determinant of the DBP response to the rhythmic handgrip exercise during PEMI in this study. The diagnostic criteria for hypertension in this study were SBP > 140 mm Hg or DBP > 90 mmHg (Umemura et al., 2019). The average resting SBP in the HT group was 147 mmHg, which met the criteria for hypertension; however, the resting average DBP (89 mmHg) did not meet the criteria for hypertension. Therefore, resting SBP, which exceeded the criteria, may have been a significant factor influencing the DBP response to the rhythmic handgrip exercise during PEMI in this study.

Finally, although significant differences were observed in the BP response to the rhythmic handgrip exercise during PEMI between the groups, no group differences were found in the HR response. This may be because, in addition to the sympathetic nervous system, the parasympathetic nervous system also innervates the heart, and some central regulatory mechanisms differ from those of BP (Teixeira et al., 2020).

### 4.2 Limitations and strengths

We acknowledge the following limitations of this study. First, we did not continuously measure BP; therefore, any group differences in detailed hemodynamics, including peak values, during stimulations were not clarified. Second, the relatively small sample size, while meeting the minimum requirement determined through an a priori power analysis, may not accurately represent the population, leading to biased results or overestimating the effects. Third, as mentioned above, we did not directly assess MSNA, which limits consideration of the detailed neural mechanisms underlying the enhanced BP response to ischemic dynamic muscle contraction in hypertension. Fourth, although BP regulation during exercise is affected by sex hormones (Choi et al., 2012; Smith et al., 2019), no information was found on hormone replacement in female participants. Finally, this study could not mention the exact interaction between hypertension and aging because young and middle‐aged patients with hypertension were omitted.

Despite these limitations, this study has several notable strengths. First, most importantly, the additional multivariate analysis identified resting BP as a significant independent determinant of BP responses to ischemic dynamic exercise, while the potential confounding effects of sex, muscle strength, and BMI cannot be excluded. Second, although many studies have examined the effects of hypertension on cardiovascular responses to ischemic dynamic exercise, including blood flow‐restricted resistance exercise (Araújo et al., 2014; Barili et al., 2018; Cezar et al., 2016; Chulvi‐Medrano, 2015; Pinto et al., 2018; Pinto & Polito, 2016; Zaboli et al., 2024; Zhao et al., 2022), most have assessed these responses only before and after exercise (Araújo et al., 2014; Barili et al., 2018; Cezar et al., 2016; Chulvi‐Medrano, 2015; Zaboli et al., 2024; Zhao et al., 2022), rather than during exercise itself (Pinto et al., 2018; Pinto & Polito, 2016). Additionally, to the best of our knowledge, few studies have focused exclusively on older adults (Barili et al., 2018; Pinto et al., 2018), and none have compared pressor responses during ischemic dynamic exercise between the hypertensive and normotensive groups. Therefore, this study is unique because it specifically investigates the effects of hypertension on BP responses “during” ischemic dynamic exercise in “older adults.” Third and most importantly, ischemia (Cornett et al., 2000; Cui et al., 2008), aging (Daida et al., 1996; Milia et al., 2015; Trinity et al., 2018), and hypertension (Chulvi‐Medrano, 2015; Dombrowski et al., 2018; Laterza et al., 2007; Leal et al., 2008; Liang et al., 2016; Mitchell, 2017; Mizuno et al., 2011a; Seals et al., 1985; Sidhu et al., 2019; Smith, Williams, et al., 2006) are known to enhance the exercise pressor response independently. Together with our previous study (Hasegawa et al., 2021), the present study suggests for the first time, to the best of our knowledge, that these factors additively enhance BP response to exercise, although they may redundantly mask each other in some respects.

### 4.3 Perspectives and significance

Our study demonstrates that hypertension exacerbates the pressor response to ischemic dynamic exercise in older adults. Ischemia‐like physical activities, including climbing long flights of stairs, are common in daily life and can create an imbalance between oxygen supply and demand. Older adults with resting SBP > 150 mmHg, similar to that of participants in this study, face increased risks of cardiovascular events (Masoli et al., 2020). An excessive BP response during physical activities further elevates the risk of cardiovascular events (Al Ghorani et al., 2022; Cho et al., 2012). Therefore, while careful consideration is needed when applying these findings to clinical practice, this study raises significant concerns regarding the heightened cardiovascular risk during physical activity in older individuals with hypertension.

## 5 CONCLUSION

This study showed that hypertension intensified the BP response to ischemic dynamic exercise in adults aged ≥60 years. These results suggest that hypertension exacerbates the enhanced BP response to ischemic dynamic exercise in older adults.

## AUTHOR CONTRIBUTIONS

DH and NH conceived and designed the research; DH, AH, YO, and NH performed the experiments; DH, AH, KS, and NH analyzed the data; DH, AH, KS, MM, and NH interpreted the results of the experiments; DH and NH prepared the figures; DH, MM, and NH drafted the manuscript; DH, MM, and NH edited and revised the manuscript; All authors approved the manuscript.

## FUNDING INFORMATION

This work was supported by the Health Science Center Foundation grant number 201704 (to NH) and JSPS KAKENHI grant number 22KK0154 (to NH).

## CONFLICT OF INTEREST STATEMENT

No conflicts of interest to declare.

## ETHICS STATEMENT

This study was approved by the Ethics Committee of Chubu University (No. 290077–3) and conformed to the principles of the Declaration of Helsinki. The study adhered to the institutional guidelines and regulations for all procedures performed. All participants provided written consent after they were informed about the experimental protocol, including the potential benefits and risks.

## Supporting information


Appendix S1.

## Data Availability

The datasets generated during and/or analyzed during the current study are available from the corresponding author upon reasonable request.
