# Supplementary material for: Does hypertension exacerbate the age‐related exaggerated pressor response to dynamic exercise during post‐exercise muscle ischemia?
Source: Physiol Rep. 2025 Jun 6;13(11):e70365. doi: 10.14814/phy2.70365 (PMC12141974; doi:10.14814/phy2.70365)
Supplement: Supplementary file 1 — Appendix S1. [file PHY2-13-e70365-s001.docx]

**SUPPLEMENTAL MATERIALS**

**Does hypertension exacerbate the age-related exaggerated pressor response to dynamic exercise during post-exercise muscle ischemia?**

Daisuke Hasegawa,^1^ Amane Hori,^1,2,3^ Yukiko Okamura,^1^ Kenichi Suijo,^1^ Masaki Mizuno,^3^ Norio Hotta^1^

^1^ College of Life and Health Sciences, Chubu University, Kasugai, Aichi, Japan

^2^ Japan Society for the Promotion of Science, Chiyoda-ku, Tokyo, Japan

^3^ Department of Applied Clinical Research, UT Southwestern Medical Center, Dallas, Texas, USA

**Address for correspondence:**

Norio Hotta, Ph.D.

College of Life and Health Sciences, Chubu University

Matsumoto-cho 1200, Kasugai, Aichi 487-8501, Japan

E-mail: horinori@isc.chubu.ac.jp

Tel: (81) 568-51-9667

**Supplemental Table S1. Post hoc comparisons of circulatory responses to each stimulus mode versus the baseline in hypertensive (HT) and non-hypertensive (NHT) groups from Figure 1**

Significant p-values are indicated in bold. SBP, systolic blood pressure; DBP, diastolic blood pressure; HR, heart rate; PEMI, post-exercise muscle ischemia. Tukey’s test was employed for post-hoc analysis.


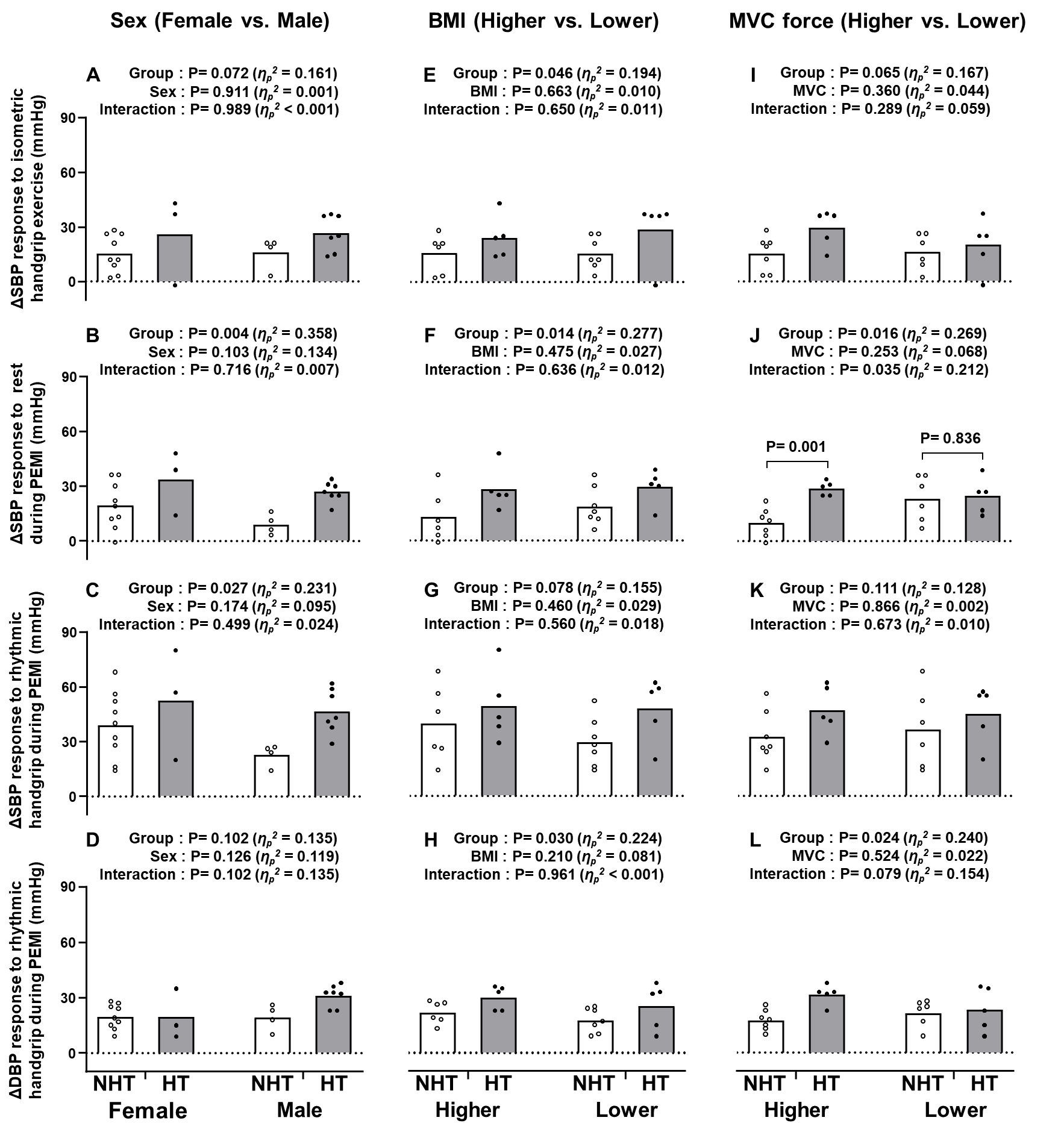


**Supplemental Fig. S1. Influence of sex, BMI, and MVC force on the effects of hypertension on blood pressure responses (Δ) to isometric handgrip exercise, as well as rest and rhythmic handgrip exercise during post-exercise muscle ischemia (PEMI) in older adults**

NHT, nonhypertensive; HT, hypertensive; SBP, systolic blood pressure; DBP, diastolic blood pressure; MVC, maximal handgrip strength; BMI, body mass index. Data were tested using a two-factor ANOVA. *η_p_*^2^ was used as the effect size (0.01, 0.06, and 0.14 represent small, medium, and large, respectively). Tukey’s test was employed for post-hoc analysis. Values are means. Participants were categorized based on sex (female vs. male, A–D), BMI (higher vs. lower groups, E–H), and MVC force (higher vs. lower groups, I–L). Blood pressure response indices that showed significant group differences in Figure 1 were chosen. The NHT older adults were selected from our previous study (Hasegawa et al., 2021).

**Supplemental Table S2.** **Multivariable-adjusted linear regression models with BMI included as an additional covariate**

Body mass index (BMI) was included as an additional covariate in the multiple regression analyses presented in Table 6. The second model—systolic blood pressure (SBP) response to rhythmic handgrip during post-exercise muscle ischemia (PEMI)—is identical to that in Table 6, as BMI had already been included as an independent variable. In the third model—diastolic blood pressure (DBP) response to rhythmic handgrip during PEMI—sex was excluded as a covariate due to multicollinearity (variance inflation factor [VIF] > 5.0). All remaining VIFs were < 1.48. R^2^ and β represent the adjusted coefficient of determination and the standardized regression coefficient, respectively. MVC, maximal handgrip strength. n = 23.
